# Editorial: Neuroendocrine regulation of feeding and reproduction in fish

**DOI:** 10.3389/fendo.2023.1160378

**Published:** 2023-02-17

**Authors:** Bin Wang, Shan He, José A. Muñoz-Cueto

**Affiliations:** ^1^ Key Laboratory of Sustainable Development of Marine Fisheries, Ministry of Agriculture and Rural Affairs, Yellow Sea Fisheries Research Institute, Chinese Academy of Fishery Sciences, Qingdao, China; ^2^ Laboratory for Marine Fisheries and Food Production Processes, Pilot National Laboratory for Marine Science and Technology (Qingdao), Qingdao, China; ^3^ College of Fisheries, Huazhong Agricultural University, Wuhan, China; ^4^ Engineering Research Center of Green Development for Conventional Aquatic Biological Industry in the Yangtze River Economic Belt, Ministry of Education, Wuhan, China; ^5^ Department of Biology, Faculty of Marine and Environmental Sciences, University of Cádiz, Cádiz, Spain; ^6^ Marine Research Institute (INMAR), Marine Campus of International Excellence (CEIMAR) and Agrifood Campus of International Excellence (ceiA3), Cádiz, Spain; ^7^ The European University of the Seas (SEA-EU), Cádiz, Spain

**Keywords:** feeding, reproduction, neuroendocrine factors, teleosts, aquaculture

The appropriate choice and intake of food and nutrients have a great beneficial impact on health, growth, reproduction and lifespan of fish. Nevertheless, nutritional requirements vary according to the life cycle, physiological and reproductive status of animals. Feeding is a complex behavior regulated by central and peripheral neuroendocrine/endocrine signals, in which hypothalamic neuroendocrine factors such as orexin, galanin, neuropeptide Y (NPY), agouti-related peptide (AgRP), proopiomelanocortin (POMC), cocaine- and amphetamine-regulated transcript (CART), among others, could play an important role (1–3). In addition to the “classical” neuroendocrine factors, e.g., gonadotropin-releasing hormone (GnRH), dopamine, γ-aminobutyric acid (GABA), serotonin and NPY, new actors as gonadotropin-inhibitory hormone (GnIH), kisspeptin (Kiss), spexin (SPX), neurokinin B/tachykinin (NKB/TAC) and secretoneurin (SN) have gained increasing importance in the regulation of fish reproduction over the last decade (4–13).

It is interesting to note that some of these neuroendocrine factors could modulate food intake as well as gonadal development/maturation and reproductive performance through complex interactions along the hypothalamic-pituitary-gonadal (HPG) axis (5, 14–16). The main objective of the present Research Topic is to provide a comprehensive and updated vision on the neuroendocrine control of feeding and reproduction in fish, focusing especially on the factors that could modulate both physiological processes. This Research Topic contains 13 contributions, 9 original research articles and 4 review papers, which reported recent advances in physiological actions, signaling pathways, evolution and transcriptional regulation of neuroendocrine systems modulating feeding and reproduction in fish.

Four manuscripts in the present Research Topic focused on the role of kisspeptins in fish reproduction and the interaction of stress and reproductive axes. The first review paper by Wang et al. summarized the research progresses of kisspeptin and its receptors (KissR) in teleost fish, with particular emphasis on molecular diversity, phylogenetic evolution, tissue distribution, physiological actions on reproduction, intracellular signaling pathways and regulatory mechanisms. They also highlighted some relevant aspects of the kisspeptinergic system in flatfish species. Predicted *kiss1* and *kissr3* gene sequences are found in the genomes of Senegalese sole, half-smooth tongue sole and turbot, which were previously thought to be lost during evolution in Pleuronectiformes (17, 18). Zhao et al. reported their investigation on the identification and physiological roles of kisspeptins (Kiss1, Kiss2) and their receptors (KissR2 and KissR3) in the control of reproduction in turbot. They confirmed the existence of *kiss1*/*kissr3* genes in this flatfish species by molecular cloning, and showed that both Kiss1 and Kiss2 stimulated pituitary gonadotropin gene expression. Zahangir et al. found in their original research article that Kiss2 significantly stimulated the expression of *kissr2*, *gnrh1* and gonadotropin subunits in a gonadal stage-dependent manner in male grass puffer, which has only a single pair of *kiss2* and *kissr2* (19). In addition, Bu et al. described the molecular mechanisms of glucocorticoid regulation of *kiss1* and *kiss2* genes in yellowtail clownfish. Cortisol stimulated mRNA levels of *kiss1*, *kiss2*, glucocorticoid receptor 1 (*gr1*) and 2 (*gr2*). Particularly, cortisol enhanced *kiss2* promoter activities *via* GRs, with GR1 being more effective than GR2. These data provide additional evidence for the involvement of kisspeptin in the regulation of stress-induced reproductive disorders.

The tachykinin/neurokinin B family of neuropeptides was addressed in a review manuscript and an original paper. The second review paper of this Research Topic is authored by Campo et al., and compared the similarities and differences of tachykinin systems and biological roles in the control of reproduction and food intake between mammals and teleosts. In humans, the tachykinin system comprises three *tac* genes (*tac1*, *tac3*, and *tac4*) which encode 10 different mature peptides and three TAC receptors (TACR1, TACR2, and TACR3). In fish, however, duplicates for *tac1* (*tac1a* and *tac1b*), *tac3* (*tac3a* and *tac3b*), and *tac4* (*tac4a* and *tac4b*) exist and 12 different mature peptides have been identified so far. In turn, up to six TACR types have been characterized in fish: two TACR1 (TACR1a and TACR1b), one TACR2, and three TACR3 (TACR3a1, TACR3a2, and TACR3b). In contrast to mammals, TAC3 peptides (NKB and NKBRP) have various effects on reproduction in teleosts, mainly depending on the species, the maturity stage, and the peptide tested. Further studies are urgently needed to clarify the direct actions of TAC peptides on the expression of central actors involved in the control of food intake in both mammals and teleosts. Zuo et al. investigated the reproductive function of the TAC3/TACR3 system in a catadromous teleost, the Japanese eel. They found that two *tac3* (*tac3a* and *tac3b*) and one *tacr3* (*tacr3a*) genes exist in this species, and confirmed that a mutation caused early termination of TACR3 protein, resulting in the loss of 35 amino acids at the C-terminal of the receptor. Thus, neither NKB nor NKBRP peptides encoded by *tac3* genes could increase CRE-luc and SRE-luc activities *via* their cognate receptor. However, NKB significantly stimulated *gnrh1*, *fshb* or *lhb* mRNA levels, perhaps *via* other receptors.

Two additional manuscripts of this Research Topic focused on hypothalamo-neurohypophyseal neuropeptides. The third review paper by Mennigen et al. discussed the reproductive physiology of the arginine vasopressin (AVP)/oxytocin (OXT) neuropeptide family in teleost fishes. They reviewed the current state of knowledge regarding this teleost nonapeptide system, such as structure, evolution, anatomy, receptor repertoire, regulation, among others. This article mainly focused on the reproductive function, with emphasis on reproductive behavior, reproductive cues, and actions on the HPG axis. Of note, nonapeptide homologues of AVP and OXT in bony fish are designated as vasotocin (VT) and isotocin (IT), respectively. Zhang et al. reported in pregnant lined seahorse that injection of VT intraperitoneally induced premature parturition, and up-regulated serum estrogen concentration and transcript levels of pituitary *fshb*/*lhb* and brood pouch G protein-coupled estrogen receptor (*gper*), however, down-regulated pituitary prolactin (*prl*) mRNA levels. These results suggest that VT could promote premature parturition of seahorse by regulating estrogen synthesis through the HPG axis.

The fourth review paper by Assan et al. is centered on advancements in food intake and feeding behavior regulation in fish, dietary selection and preference and the influence of some extrinsic factors, such as stress, temperature, hypoxia, photoperiod/light regime, circannual and circadian rhythms. They highlighted the physiological role of apelin, a novel appetite-regulating peptide (3), in the modulation of feeding, along with response to different nutritional status in various fish species. It has been well documented that GnRH is a highly conserved decapeptide that is essential for reproduction in vertebrates, but recent *gnrh* knockout studies in zebrafish and medaka suggest that the GnRH system may not be the sole “master” of reproduction in these two species, with its role apparently being less central than originally thought (11, 20). In their original research article, Li et al. proposed GnRH as a coupling factor to integrate the feeding metabolism and reproduction in teleosts. Both GnRH2 and GnRH3 significantly stimulated mRNA levels of pituitary reproduction-related genes (*ghta*, *lhb*, *fshb*, *inhba*, and *sg2*) through the AC/PKA, PLC/IP3/PKC, and Ca^2+^/CaM/CaMK-II pathways, but reduced dopamine receptor 2 (*drd2*) gene expression *via* the Ca^2+^/CaM/CaMK-II pathway. In addition, these two neuropeptides also enhanced transcript abundance of pituitary anorexigenic peptides (*pomcb*, *cart2*, *uts1*, *nmba*, and *nmbb*) through the AC/PKA, PLC/IP3/PKC, and Ca^2+^/CaM/CaMK-II pathways. In turn, Ren et al. provided evidence in pompano for the participation of GnRH1 in the immune regulation of liver disease and the regulation of digestive and metabolic enzyme activities, suggesting that GnRH1 has also non reproductive related functions. Taken together, GnRH seems to be a candidate for the integration of reproduction and metabolism in fish.

Since the first discovery of gonadotropin-inhibitory hormone in the quail, the presence of GnIH orthologs has been reported in a variety of vertebrate species, including fish (12). Despite its functional significance and diversity, little information is available regarding the mode of GnIH action on target cells and the potential interaction with other neuroendocrine factors (21, 22). Wang et al. elucidated the intracellular signaling pathways mediating in sea bass GnIH actions and the interactions with sea bass kisspeptin signaling. They found that sea bass GnIHR signals can be transduced through the PKA and PKC pathways, and GnIH can interfere with kisspeptin actions by reducing its signaling. On the other hand, spexin 1 (SPX1) has recently emerged as a neuropeptide with pleotropic functions in vertebrates, including the regulation of feeding and reproduction, but knowledge about the physiological role or biological action of SPX2 is still very limited in fish (6, 7). In this Research Topic, Wang et al. evaluated the effects of intraperitoneal injection of endogenous SPX2 on the expression levels of reproductive genes of the brain-pituitary axis in half-smooth tongue sole, providing pioneer evidence for the involvement of SPX2 in the regulation of reproduction in any vertebrate. The functional significance of SPX2, which only exists in non-mammalian species, warrant more investigation in depth.

Finally, we would like to express our most sincere thanks to the authors, the reviewers, and the journal staff for their efforts to make this Research Topic so fruitful.

## Author contributions

BW and SH wrote the manuscript. JAM-C edited the manuscript. All authors contributed to the article and approved the submitted version.
