# Corrigendum: Editorial: Neuroendocrine regulation of feeding and reproduction in fish

**DOI:** 10.3389/fendo.2023.1215915

**Published:** 2023-08-15

**Authors:** Bin Wang, Shan He, José A. Muñoz-Cueto

**Affiliations:** ^1^ Key Laboratory of Sustainable Development of Marine Fisheries, Ministry of Agriculture and Rural Affairs, Yellow Sea Fisheries Research Institute, Chinese Academy of Fishery Sciences, Qingdao, China; ^2^ Laboratory for Marine Fisheries and Food Production Processes, Pilot National Laboratory for Marine Science and Technology (Qingdao), Qingdao, China; ^3^ College of Fisheries, Huazhong Agricultural University, Wuhan, China; ^4^ Engineering Research Center of Green Development for Conventional Aquatic Biological Industry in the Yangtze River Economic Belt, Ministry of Education, Wuhan, China; ^5^ Department of Biology, Faculty of Marine and Environmental Sciences, University of Cádiz, Cádiz, Spain; ^6^ Marine Research Institute (INMAR), Marine Campus of International Excellence (CEIMAR) and Agrifood Campus of International Excellence (ceiA3), Cádiz, Spain; ^7^ The European University of the Seas (SEA-EU), Cádiz, Spain

**Keywords:** feeding, reproduction, neuroendocrine factors, teleosts, aquaculture

In the published article, there was an error in the Funding statement. The Funding statement was displayed as “This work was supported by grants from National Natural Science Foundation of China 32072949 to BW, 32172951 to SH, PAIDI2020 (Consejería de Economía, Conocimiento, Empresas y Universidad. Junta de Andalucía. Grant no P18-RT-5152) to JAM-C”. The correct Funding statement appears below.

**FUNDING**

“This work was supported by grants from National Natural Science Foundation of China 32072949 to BW, 32172951 to SH, PAIDI2020 (Consejería de Economía, Conocimiento, Empresas y Universidad. Junta de Andalucía. Grant no P18-RT-5152) and FEDER-UCA (Grant no 18-107538) to JAM-C.”

The authors apologize for this error and state that this does not change the scientific conclusions of the article in any way. The original article has been updated.

